# 4-Benzyl-6-bromo-2-(4-methoxy­phen­yl)-4*H*-imidazo[4,5-*b*]pyridine monohydrate

**DOI:** 10.1107/S1600536810010391

**Published:** 2010-03-27

**Authors:** Y. Ouzidan, Y. Kandri Rodi, S. Obbade, El Mokhtar Essassi, Seik Weng Ng

**Affiliations:** a Laboratoire de Chimie Organique Appliquée, Faculté des Sciences et Techniques, Université Sidi Mohamed Ben Abdallah, Fés, Morocco; b Laboratoire d’Electrochimie et de Physicochimie des Matériaux et des Interfaces, A313 Domaine Universitaire, 38402 St Martin d’Hères, Grenoble, France; c Laboratoire de Chimie Organique Hétérocyclique, Pôle de Compétences Pharmacochimie, Université Mohammed V-Agdal, BP 1014 Avenue Ibn Batout, Rabat, Morocco; d Department of Chemistry, University of Malaya, 50603 Kuala Lumpur, Malaysia

## Abstract

The imidazopyridine fused ring in the title compound, C_20_H_16_BrN_3_O·H_2_O, is coplanar with the aromatic ring at the 2-position [dihedral angle = 5.2 (1)°]. In the five-membered imidazo portion, the C—N bond whose C atom is also connected to the pyridine N atom has predominantly double-bond character [1.334 (2) Å] whereas the C—N bond whose atom is connected to the pyridine C atom has predominantly single-bond character [1.371 (2) Å]. The water mol­ecule engages in hydrogen bonding with the latter N atom; it is also connected to a symmetry-related water mol­ecule, generating a linear chain structure.

## Related literature

For the crystal structure of 4-benzyl-6-bromo-2-phenyl-4*H*-imidazo[4,5-*b*]pyridine, see: Ouzidan *et al.* (2010 ▶).
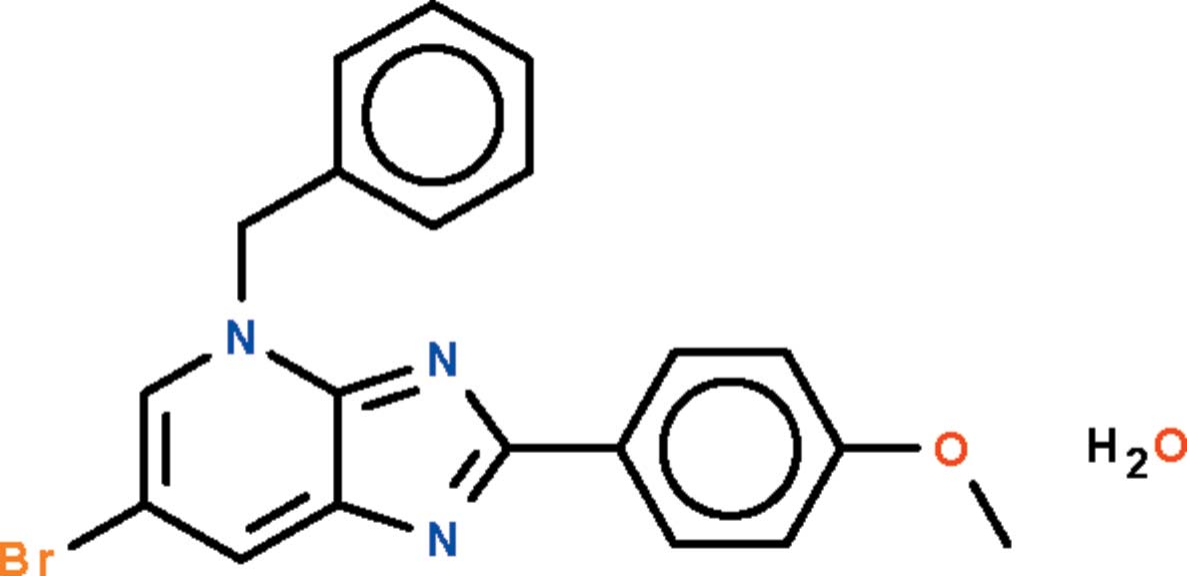


## Experimental

### 

#### Crystal data

C_20_H_16_BrN_3_O·H_2_O
*M* *_r_* = 412.28
Monoclinic, 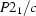

*a* = 10.5924 (2) Å
*b* = 5.4544 (1) Å
*c* = 31.7444 (7) Å
β = 100.292 (1)°
*V* = 1804.53 (6) Å^3^
*Z* = 4
Mo *K*α radiation
μ = 2.30 mm^−1^
*T* = 293 K
0.29 × 0.13 × 0.09 mm

#### Data collection

Bruker X8 APEXII diffractometer
Absorption correction: multi-scan (*SADABS*; Sheldrick, 1996 ▶) *T*_min_ = 0.556, *T*_max_ = 0.820
25327 measured reflections
5183 independent reflections
3751 reflections with *I* > 2σ(*I*)
*R*_int_ = 0.029

#### Refinement

*R*[*F* ^2^ > 2σ(*F* ^2^)] = 0.035
*wR*(*F* ^2^) = 0.098
*S* = 1.02
5183 reflections
244 parameters
2 restraints
H atoms treated by a mixture of independent and constrained refinement
Δρ_max_ = 0.32 e Å^−3^
Δρ_min_ = −0.38 e Å^−3^

### 

Data collection: *APEX2* (Bruker, 2008 ▶); cell refinement: *SAINT* (Bruker, 2008 ▶); data reduction: *SAINT*; program(s) used to solve structure: *SHELXS97* (Sheldrick, 2008 ▶); program(s) used to refine structure: *SHELXL97* (Sheldrick, 2008 ▶); molecular graphics: *X-SEED* (Barbour, 2001 ▶); software used to prepare material for publication: *publCIF* (Westrip, 2010 ▶).

## Supplementary Material

Crystal structure: contains datablocks global, I. DOI: 10.1107/S1600536810010391/pk2233sup1.cif

Structure factors: contains datablocks I. DOI: 10.1107/S1600536810010391/pk2233Isup2.hkl

Additional supplementary materials:  crystallographic information; 3D view; checkCIF report

## Figures and Tables

**Table 1:** Hydrogen-bond geometry (Å, °)

*D*—H⋯*A*	*D*—H	H⋯*A*	*D*⋯*A*	*D*—H⋯*A*
O1w—H11⋯N2	0.85 (1)	2.30 (2)	3.092 (3)	155 (5)
O1w—H12⋯O1*W*^i^	0.85 (1)	2.30 (2)	3.119 (2)	162 (5)

Symmetry code: (i) 
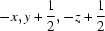
.

**Fig. 1.**
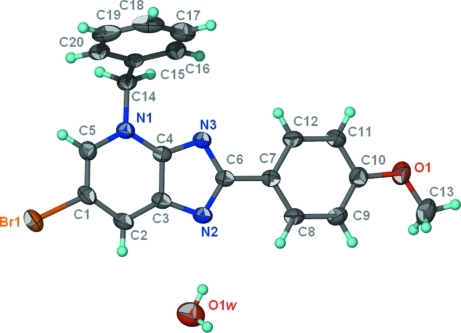
Thermal ellipsoid plot (Barbour, 2001) of C20H16BrN3O.H2O at the 50% probability level; hydrogen atoms are drawn as spheres of arbitrary radius.

## supplementary crystallographic information

### Comment

Imidazo[4,5-*b*]pyridines are a class of sedative drugs. In the previous study, we reacted 6-bromo-2-phenyl-1*H*-imidazo[4,5-*b*]pyridine with benzyl chloride in the presence of a catalytic quantity of tetra-*n*-butylammonium bromide under mild conditions to form 4-benzyl-6-bromo-2-phenyl-4*H*-imidazo[4,5-*b*]pyridine (Ouzidan *et al.*, 2010). The study is extended to the synthesis of the 2(4-methoxyphenyl) analog to furnish the title hydrate (Scheme I, Fig. 1). The imidazopyridine fused-ring in the C_20_H_16_BrN_3_O molecule is co-planar with the aromatic ring at the 2-position [dihedral angle 5.2 (1) °]. In the five-membered imidazo portion, the carbon–nitrogen bond whose carbon atom is also connected to the pyridine nitrogen atom is a double bond [1.334 (2) Å] whereas the carbon–nitrogen bond whose atom is connected to the pyridine carbon atom is a single bond [1.371 (2) Å]. The water molecule engages in hydrogen bonding with the latter nitrogen atom; it is also connected to a symmetry-related water molecule to generate a linear chain structure.

### Experimental

To a solution of the 6-bromo-2-(4-methoxyphenyl)-1*H*-imidazo[4,5-*b*]pyridine (0.33 g, 1.21 mmol), potassium carbonate (0.20 g, 1.42 mmol) and tetra-*n*-butylammonium bromide (0.04 g (0,1 mmol) in DMF (15 ml) was added benzyl chloride (0.15 ml, 1.31 mmol). Stirring was continued at room temperature for 12 hours. The salt was removed by filtration and the filtrate concentrated under reduced pressure. The residue was chromatographed on a column of silica gel with ethyl acetate/hexane (1/1) as eluent. Yellow crystals were isolated when the solvent was allowed to evaporate.

### Refinement

Carbon-bound H-atoms were placed in calculated positions (C—H 0.93–0.97 Å) and were included in the refinement in the riding model approximation, with *U*(H) set to 1.2*U*(C). The water H-atoms were located in a difference Fourier map, and were refined with a distance restraint of O–H 0.84 (1) Å.

### Crystal data

**Table:**

C_20_H_16_BrN_3_O·H_2_O	*F*(000) = 840
*M**_r_* = 412.28	*D*_x_ = 1.518 Mg m^−^^3^
Monoclinic, *P*2_1_/*c*	Mo *K*α radiation, λ = 0.71073 Å
Hall symbol: -P 2ybc	Cell parameters from 6654 reflections
*a* = 10.5924 (2) Å	θ = 3.0–28.2°
*b* = 5.4544 (1) Å	µ = 2.30 mm^−^^1^
*c* = 31.7444 (7) Å	*T* = 293 K
β = 100.292 (1)°	Prism, yellow
*V* = 1804.53 (6) Å^3^	0.29 × 0.13 × 0.09 mm
*Z* = 4	

### Data collection

**Table:**

Bruker X8 APEXII diffractometer	5183 independent reflections
Radiation source: fine-focus sealed tube	3751 reflections with *I* > 2σ(*I*)
graphite	*R*_int_ = 0.029
φ and ω scans	θ_max_ = 29.8°, θ_min_ = 3.0°
Absorption correction: multi-scan (*SADABS*; Sheldrick, 1996)	*h* = −14→14
*T*_min_ = 0.556, *T*_max_ = 0.820	*k* = −7→7
25327 measured reflections	*l* = −40→44

### Refinement

**Table:**

Refinement on *F*^2^	Primary atom site location: structure-invariant direct methods
Least-squares matrix: full	Secondary atom site location: difference Fourier map
*R*[*F*^2^ > 2σ(*F*^2^)] = 0.035	Hydrogen site location: inferred from neighbouring sites
*wR*(*F*^2^) = 0.098	H atoms treated by a mixture of independent and constrained refinement
*S* = 1.02	*w* = 1/[σ^2^(*F*_o_^2^) + (0.0469*P*)^2^ + 0.4879*P*] where *P* = (*F*_o_^2^ + 2*F*_c_^2^)/3
5183 reflections	(Δ/σ)_max_ = 0.001
244 parameters	Δρ_max_ = 0.32 e Å^−^^3^
2 restraints	Δρ_min_ = −0.38 e Å^−^^3^

### Fractional atomic coordinates and isotropic or equivalent isotropic displacement parameters (Å^2^)

**Table:**

	*x*	*y*	*z*	*U*_iso_*/*U*_eq_	
Br1	0.33516 (2)	0.25313 (4)	0.229786 (7)	0.06505 (10)	
O1W	0.0347 (2)	1.0524 (4)	0.27301 (7)	0.0809 (5)	
H11	0.091 (3)	1.063 (10)	0.2955 (9)	0.18 (2)*	
H12	0.032 (5)	1.199 (4)	0.2641 (17)	0.16 (2)*	
N1	0.49030 (13)	0.4485 (3)	0.35254 (4)	0.0370 (3)	
N2	0.25772 (13)	0.9195 (3)	0.34637 (4)	0.0397 (3)	
N3	0.43173 (14)	0.7798 (2)	0.39573 (5)	0.0368 (3)	
O1	0.24437 (17)	1.6309 (3)	0.50907 (5)	0.0663 (4)	
C1	0.36408 (17)	0.4170 (3)	0.28302 (5)	0.0437 (4)	
C2	0.28596 (17)	0.6138 (4)	0.28937 (5)	0.0438 (4)	
H2	0.2193	0.6666	0.2682	0.053*	
C3	0.31304 (16)	0.7260 (3)	0.32892 (6)	0.0380 (4)	
C4	0.41915 (15)	0.6437 (3)	0.36032 (5)	0.0351 (3)	
C5	0.46265 (17)	0.3344 (3)	0.31392 (6)	0.0417 (4)	
H5	0.5108	0.1995	0.3084	0.050*	
C6	0.33092 (15)	0.9407 (3)	0.38565 (5)	0.0355 (3)	
C7	0.30567 (16)	1.1232 (3)	0.41692 (5)	0.0374 (3)	
C8	0.20011 (19)	1.2782 (3)	0.40877 (6)	0.0466 (4)	
H8	0.1446	1.2678	0.3826	0.056*	
C9	0.17580 (19)	1.4476 (4)	0.43868 (6)	0.0501 (5)	
H9	0.1041	1.5485	0.4327	0.060*	
C10	0.25837 (19)	1.4669 (3)	0.47759 (6)	0.0462 (4)	
C11	0.3644 (2)	1.3137 (4)	0.48642 (6)	0.0501 (5)	
H11A	0.4197	1.3246	0.5126	0.060*	
C12	0.38781 (18)	1.1462 (4)	0.45650 (5)	0.0448 (4)	
H12A	0.4597	1.0458	0.4626	0.054*	
C13	0.1389 (3)	1.7962 (4)	0.50100 (10)	0.0722 (7)	
H13A	0.1424	1.9056	0.5249	0.108*	
H13B	0.0601	1.7054	0.4971	0.108*	
H13C	0.1431	1.8891	0.4756	0.108*	
C14	0.60115 (17)	0.3669 (3)	0.38527 (6)	0.0409 (4)	
H14A	0.6095	0.1901	0.3839	0.049*	
H14B	0.5856	0.4091	0.4136	0.049*	
C15	0.72405 (16)	0.4858 (3)	0.37807 (5)	0.0376 (4)	
C16	0.76570 (19)	0.7021 (3)	0.39913 (6)	0.0460 (4)	
H16	0.7194	0.7703	0.4185	0.055*	
C17	0.8757 (2)	0.8163 (4)	0.39140 (8)	0.0592 (5)	
H17	0.9033	0.9607	0.4057	0.071*	
C18	0.9443 (2)	0.7187 (5)	0.36287 (8)	0.0675 (7)	
H18	1.0174	0.7983	0.3574	0.081*	
C19	0.9054 (2)	0.5027 (5)	0.34216 (7)	0.0644 (7)	
H19	0.9529	0.4356	0.3230	0.077*	
C20	0.79559 (18)	0.3847 (4)	0.34969 (6)	0.0495 (5)	
H20	0.7698	0.2382	0.3358	0.059*	

### Atomic displacement parameters (Å^2^)

**Table:**

	*U*^11^	*U*^22^	*U*^33^	*U*^12^	*U*^13^	*U*^23^
Br1	0.06531 (16)	0.07264 (17)	0.05167 (13)	0.00232 (11)	−0.00455 (10)	−0.02553 (10)
O1W	0.0702 (12)	0.0877 (14)	0.0805 (13)	−0.0048 (10)	0.0017 (10)	0.0014 (11)
N1	0.0327 (7)	0.0387 (7)	0.0383 (7)	−0.0010 (6)	0.0027 (5)	0.0004 (6)
N2	0.0325 (7)	0.0490 (8)	0.0361 (7)	0.0033 (6)	0.0020 (5)	0.0001 (6)
N3	0.0330 (7)	0.0413 (8)	0.0349 (7)	0.0013 (6)	0.0024 (5)	0.0003 (5)
O1	0.0887 (11)	0.0604 (9)	0.0474 (8)	0.0241 (9)	0.0063 (7)	−0.0108 (7)
C1	0.0387 (9)	0.0493 (10)	0.0411 (8)	−0.0084 (8)	0.0018 (7)	−0.0091 (7)
C2	0.0340 (9)	0.0543 (11)	0.0402 (8)	−0.0023 (8)	−0.0016 (7)	−0.0028 (8)
C3	0.0296 (8)	0.0457 (10)	0.0375 (8)	−0.0033 (7)	0.0025 (6)	0.0006 (7)
C4	0.0292 (8)	0.0397 (8)	0.0357 (8)	−0.0028 (7)	0.0041 (6)	0.0022 (6)
C5	0.0388 (9)	0.0396 (9)	0.0460 (9)	−0.0031 (7)	0.0061 (7)	−0.0059 (7)
C6	0.0307 (8)	0.0414 (9)	0.0340 (7)	−0.0010 (7)	0.0044 (6)	0.0027 (6)
C7	0.0354 (8)	0.0419 (9)	0.0343 (8)	0.0007 (7)	0.0049 (6)	0.0026 (7)
C8	0.0423 (10)	0.0532 (11)	0.0407 (9)	0.0102 (8)	−0.0019 (7)	−0.0005 (7)
C9	0.0486 (11)	0.0530 (11)	0.0469 (10)	0.0175 (9)	0.0036 (8)	0.0006 (8)
C10	0.0568 (11)	0.0437 (10)	0.0382 (8)	0.0056 (8)	0.0090 (8)	0.0002 (7)
C11	0.0555 (11)	0.0559 (11)	0.0347 (9)	0.0090 (9)	−0.0035 (8)	−0.0017 (8)
C12	0.0424 (10)	0.0513 (10)	0.0380 (8)	0.0099 (8)	0.0000 (7)	0.0035 (8)
C13	0.0841 (18)	0.0593 (14)	0.0763 (17)	0.0176 (12)	0.0229 (14)	−0.0141 (12)
C14	0.0414 (9)	0.0387 (9)	0.0406 (8)	0.0037 (7)	0.0015 (7)	0.0072 (7)
C15	0.0337 (8)	0.0409 (9)	0.0359 (8)	0.0094 (7)	−0.0001 (6)	0.0084 (6)
C16	0.0436 (10)	0.0443 (10)	0.0477 (10)	0.0036 (8)	0.0020 (8)	0.0036 (7)
C17	0.0487 (12)	0.0578 (12)	0.0652 (13)	−0.0073 (10)	−0.0060 (10)	0.0126 (10)
C18	0.0383 (11)	0.097 (2)	0.0640 (14)	−0.0018 (11)	−0.0006 (10)	0.0325 (13)
C19	0.0426 (11)	0.107 (2)	0.0454 (10)	0.0293 (12)	0.0118 (9)	0.0190 (12)
C20	0.0471 (11)	0.0600 (12)	0.0386 (9)	0.0204 (9)	0.0006 (8)	0.0025 (8)

### Geometric parameters (Å, °)

**Table:**

Br1—C1	1.8877 (17)	C9—C10	1.384 (3)
O1w—H11	0.85 (1)	C9—H9	0.9300
O1w—H12	0.85 (1)	C10—C11	1.388 (3)
N1—C4	1.352 (2)	C11—C12	1.372 (3)
N1—C5	1.359 (2)	C11—H11A	0.9300
N1—C14	1.490 (2)	C12—H12A	0.9300
N2—C6	1.351 (2)	C13—H13A	0.9600
N2—C3	1.371 (2)	C13—H13B	0.9600
N3—C4	1.334 (2)	C13—H13C	0.9600
N3—C6	1.375 (2)	C14—C15	1.508 (2)
O1—C10	1.369 (2)	C14—H14A	0.9700
O1—C13	1.423 (3)	C14—H14B	0.9700
C1—C5	1.374 (3)	C15—C16	1.389 (3)
C1—C2	1.392 (3)	C15—C20	1.390 (3)
C2—C3	1.380 (2)	C16—C17	1.381 (3)
C2—H2	0.9300	C16—H16	0.9300
C3—C4	1.435 (2)	C17—C18	1.366 (4)
C5—H5	0.9300	C17—H17	0.9300
C6—C7	1.464 (2)	C18—C19	1.376 (4)
C7—C8	1.389 (2)	C18—H18	0.9300
C7—C12	1.400 (2)	C19—C20	1.387 (3)
C8—C9	1.382 (3)	C19—H19	0.9300
C8—H8	0.9300	C20—H20	0.9300
			
H11—O1W—H12	101 (5)	C12—C11—C10	120.05 (17)
C4—N1—C5	119.19 (14)	C12—C11—H11A	120.0
C4—N1—C14	120.23 (14)	C10—C11—H11A	120.0
C5—N1—C14	120.53 (15)	C11—C12—C7	121.39 (17)
C6—N2—C3	102.87 (13)	C11—C12—H12A	119.3
C4—N3—C6	101.69 (13)	C7—C12—H12A	119.3
C10—O1—C13	117.82 (18)	O1—C13—H13A	109.5
C5—C1—C2	123.00 (16)	O1—C13—H13B	109.5
C5—C1—Br1	117.70 (14)	H13A—C13—H13B	109.5
C2—C1—Br1	119.29 (13)	O1—C13—H13C	109.5
C3—C2—C1	116.21 (16)	H13A—C13—H13C	109.5
C3—C2—H2	121.9	H13B—C13—H13C	109.5
C1—C2—H2	121.9	N1—C14—C15	111.09 (13)
N2—C3—C2	132.43 (16)	N1—C14—H14A	109.4
N2—C3—C4	107.37 (14)	C15—C14—H14A	109.4
C2—C3—C4	120.19 (16)	N1—C14—H14B	109.4
N3—C4—N1	128.09 (14)	C15—C14—H14B	109.4
N3—C4—C3	111.05 (15)	H14A—C14—H14B	108.0
N1—C4—C3	120.84 (15)	C16—C15—C20	119.06 (18)
N1—C5—C1	120.50 (17)	C16—C15—C14	120.06 (16)
N1—C5—H5	119.8	C20—C15—C14	120.86 (17)
C1—C5—H5	119.8	C17—C16—C15	120.2 (2)
N2—C6—N3	116.99 (15)	C17—C16—H16	119.9
N2—C6—C7	122.69 (15)	C15—C16—H16	119.9
N3—C6—C7	120.31 (14)	C18—C17—C16	120.5 (2)
C8—C7—C12	117.59 (16)	C18—C17—H17	119.8
C8—C7—C6	121.75 (15)	C16—C17—H17	119.8
C12—C7—C6	120.66 (15)	C17—C18—C19	120.1 (2)
C9—C8—C7	121.45 (17)	C17—C18—H18	120.0
C9—C8—H8	119.3	C19—C18—H18	120.0
C7—C8—H8	119.3	C18—C19—C20	120.3 (2)
C8—C9—C10	119.90 (17)	C18—C19—H19	119.9
C8—C9—H9	120.0	C20—C19—H19	119.9
C10—C9—H9	120.0	C19—C20—C15	119.9 (2)
O1—C10—C9	124.58 (17)	C19—C20—H20	120.0
O1—C10—C11	115.81 (17)	C15—C20—H20	120.0
C9—C10—C11	119.61 (17)		
			
C5—C1—C2—C3	0.4 (3)	N2—C6—C7—C12	−176.91 (17)
Br1—C1—C2—C3	−179.58 (13)	N3—C6—C7—C12	3.7 (2)
C6—N2—C3—C2	−178.68 (19)	C12—C7—C8—C9	−0.7 (3)
C6—N2—C3—C4	−0.05 (18)	C6—C7—C8—C9	179.03 (18)
C1—C2—C3—N2	−179.63 (18)	C7—C8—C9—C10	0.7 (3)
C1—C2—C3—C4	1.9 (3)	C13—O1—C10—C9	−1.2 (3)
C6—N3—C4—N1	−177.47 (17)	C13—O1—C10—C11	178.4 (2)
C6—N3—C4—C3	1.19 (18)	C8—C9—C10—O1	178.8 (2)
C5—N1—C4—N3	−179.48 (17)	C8—C9—C10—C11	−0.7 (3)
C14—N1—C4—N3	−2.2 (3)	O1—C10—C11—C12	−178.8 (2)
C5—N1—C4—C3	2.0 (2)	C9—C10—C11—C12	0.7 (3)
C14—N1—C4—C3	179.30 (15)	C10—C11—C12—C7	−0.8 (3)
N2—C3—C4—N3	−0.77 (19)	C8—C7—C12—C11	0.7 (3)
C2—C3—C4—N3	178.06 (16)	C6—C7—C12—C11	−178.99 (18)
N2—C3—C4—N1	178.00 (15)	C4—N1—C14—C15	−92.30 (18)
C2—C3—C4—N1	−3.2 (3)	C5—N1—C14—C15	84.99 (19)
C4—N1—C5—C1	0.3 (3)	N1—C14—C15—C16	93.17 (18)
C14—N1—C5—C1	−176.98 (16)	N1—C14—C15—C20	−85.35 (19)
C2—C1—C5—N1	−1.6 (3)	C20—C15—C16—C17	0.9 (3)
Br1—C1—C5—N1	178.39 (13)	C14—C15—C16—C17	−177.60 (17)
C3—N2—C6—N3	0.88 (19)	C15—C16—C17—C18	0.3 (3)
C3—N2—C6—C7	−178.50 (15)	C16—C17—C18—C19	−1.2 (3)
C4—N3—C6—N2	−1.33 (19)	C17—C18—C19—C20	0.8 (3)
C4—N3—C6—C7	178.06 (15)	C18—C19—C20—C15	0.4 (3)
N2—C6—C7—C8	3.4 (3)	C16—C15—C20—C19	−1.3 (2)
N3—C6—C7—C8	−175.95 (17)	C14—C15—C20—C19	177.23 (15)

### Hydrogen-bond geometry (Å, °)

**Table:**

*D*—H···*A*	*D*—H	H···*A*	*D*···*A*	*D*—H···*A*
O1w—H11···N2	0.85 (1)	2.30 (2)	3.092 (3)	155 (5)
O1w—H12···O1W^i^	0.85 (1)	2.30 (2)	3.119 (2)	162 (5)

Symmetry codes: (i) −*x*, *y*+1/2, −*z*+1/2.

## References

[bb1] Barbour, L. J. (2001). *J. Supramol. Chem.* **1**, 189–191.

[bb2] Bruker (2008). *APEX2* and *SAINT* Bruker AXS Inc., Madison, Wisconsin, USA.

[bb3] Ouzidan, Y., Kandri Rodi, Y., Obbade, S., Essassi, E. M. & Ng, S. W. (2010). *Acta Cryst.* E**66**, o947. 10.1107/S1600536810010391 PMC2983875 21580751

[bb4] Sheldrick, G. M. (1996). *SADABS* University of Göttingen, Germany.

[bb5] Sheldrick, G. M. (2008). *Acta Cryst.* A**64**, 112–122. 10.1107/S0108767307043930 18156677

[bb6] Westrip, S. P. (2010). *publCIF* In preparation.

